# The neuro-immune insights of itch: peripheral mechanisms and central glial contributions

**DOI:** 10.1186/s11658-025-00834-3

**Published:** 2026-01-06

**Authors:** Zhe Li, Ning Yu, Sidi Feng, Xinrui Wang, Yu-Xia Chu, Xiaowen Liu

**Affiliations:** 1 Department of Anesthesiology, China-Japan Friendship Hospital, Beijing, China; 2 Institute of Clinical Medical Sciences, China-Japan Friendship Hospital, Chinese Academy of Medical Sciences & Peking Union Medical College, Beijing, China; 3 State Key Laboratory of Common Mechanism Research for Major Diseases, Department of Human Anatomy, Histology and Embryology, Joint Laboratory of Anesthesia and Pain, Institute of Basic Medical Sciences Chinese Academy of Medical Sciences, School of Basic Medicine Peking, Neuroscience Center, Union Medical College, Beijing, China; 4 Department of Neurology, State Key Laboratory of Complex Severe and Rare Diseases, Peking Union Medical College Hospital, Chinese Academy of Medical Science and Peking Union Medical College, Beijing, China; 5 Dalian Medical University, Dalian, China; 6 Department of Pharmacy, Beijing Chaoyang Hospital, Capital Medical University, Beijing, China; 7 Department of Integrative Medicine and Neurobiology, School of Basic Medical Sciences, Institute of Acupuncture Research, Institutes of Integrative Medicine, Shanghai Key Laboratory of Acupuncture Mechanism and Acupoint Function, Shanghai Medical College, Fudan University, Shanghai, 200032 China

**Keywords:** Itch, Pruriceptors, Immune receptors, Glial cells

## Abstract

Itch is a common symptom of inflammatory, systemic, and neurological conditions and is often driven by persistent neuroinflammatory processes. This review explores the intricate mechanisms underlying itch, focusing on interactions among sensory neurons, immune mediators, and glial cells. Key peripheral pathways include activation of pruriceptors by histamine, interleukins, and chemokines, as well as inflammatory pathways dependent on Toll-like receptors (TLRs). These pathways promote the release of mediators such as interleukin-6 (IL-6) and C–C motif chemokine ligand 2 (CCL2). In the spinal cord, astrocytes and microglia contribute to itch amplification by releasing proinflammatory cytokines and activating signaling pathways such as signal transducer and activator of transcription 3 (STAT3) and TLR4. These processes drive central sensitization and facilitate the transition from acute to chronic itch in conditions such as atopic dermatitis, psoriasis, and allergic contact dermatitis. By summarizing advances in neuroimmune crosstalk and glial–neuronal interactions, this review identifies potential molecular targets for therapeutic strategies aimed at alleviating itch and improving patient outcomes.

**Graphical Abstract:**

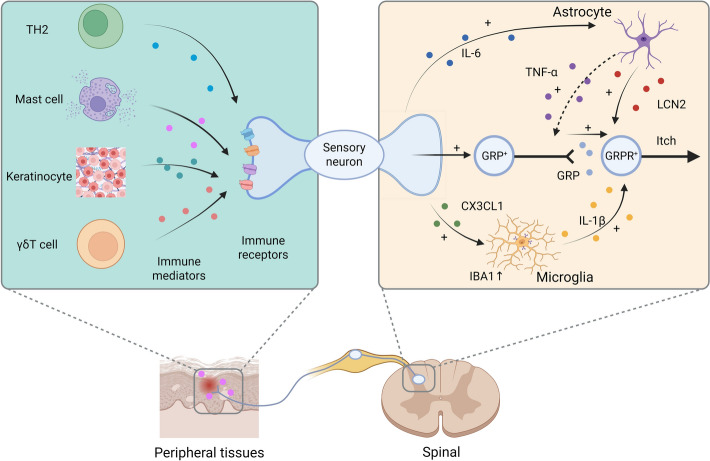

## Introduction

Itch, or pruritus, is a complex and subjective sensory experience characterized by a distinct urge to scratch. While it shares mechanistic similarities with pain in serving as an alarm system to environmental threats, itch is neurobiologically distinct from pain, operating through dedicated neural circuits and unique molecular mediators. It serves as an important biological function by alerting organisms to the presence of irritants or pathogens, but when it becomes chronic, itch can severely impair quality of life. Chronic itch, defined as pruritus persisting for more than 6 weeks, is a characteristic symptom of various skin and mucosal disorders, such as atopic dermatitis (AD), allergic conjunctivitis (ACJ), and psoriasis [1–8]. It is also closely associated with systemic conditions such as cholestasis, cutaneous T cell lymphomas, chronic liver disease, chronic kidney disease, and neuropathic diseases [9–19]. Despite its prevalence and impact, the precise mechanisms underlying itch remain incompletely understood.

Over the past decade, research has significantly advanced our understanding of the molecular and neural pathways responsible for itch [20–26]. It is now well established that pruritus is not simply a peripheral phenomenon but involves complex interactions between itch-specific neurons, immune cells, and inflammatory mediators. In chronic pruritus, neuroinflammatory processes may be dysregulated, leading to persistent activation of itch pathways even in the absence of an external trigger. This chronicity is often observed in conditions such as AD, where the interplay between inflammatory cells and the itch neural pathway creates a vicious cycle that perpetuates the itch sensation [27–30]. The process entails activation of central glial cells—including microglia and astrocytes—as well as peripheral glial and immune cells such as mast cells and T lymphocytes, which engage in crosstalk with itch-related neurons [31–33].

This review aims to summarize the neuroinflammatory mechanisms underlying itch, focusing on the crosstalk between neurons and immune cells. We examine the molecular mediators involved in this process and the neural circuits that transmit itch signals. By elucidating these mechanisms, we hope to identify potential targets for therapeutic intervention in pruritus.

## Neural mechanisms mediating itch

### Peripheral mechanisms of itch

Peripheral itch sensation originates from specialized sensory neurons located in the dorsal root ganglia (DRG) or trigeminal ganglia (TG), which innervate the skin, mucosa, and other tissues. These neurons extend peripheral terminals and are classified into Aβ, Aδ, and C fiber types on the basis of characteristic structural and electrophysiological properties. Among them, C fibers are unmyelinated and conduct impulses more slowly than myelinated Aδ and Aβ fibers. They exhibit nociceptive and pruriceptive properties and represent the primary fiber subtype involved in itch detection [34–37].

Traditionally, DRG neurons have been divided into peptidergic (PEP) and nonpeptidergic (NP) subtypes according to their neuropeptide gene-expression profiles. PEP neurons release a range of neuropeptides, including calcitonin gene-related peptide (CGRP) and substance P (SP). Despite their lower levels of neuropeptide gene expression, nonpeptidergic (NP) neurons retain the capacity to secrete distinct neuropeptides, such as neuromedin B (NMB), and are primarily composed of unmyelinated C fibers [38, 39]. While informative, this binary classification oversimplifies the molecular and functional diversity of sensory neurons.

Recent single-cell RNA sequencing studies have clarified this diversity, identifying 11 distinct types of sensory neurons [38]. These include three low-threshold mechanoreceptive neurons (LTMRs) and two proprioceptive neurons, which are largely neurofilament-rich (NF) neurons, as well as six principal types comprising thermosensitive, itch-sensitive, C-low threshold mechanoreceptors (C-LTMRs), and nociceptive neurons. Advanced genetic tools and cross-species transcriptomic atlases have further subdivided these broad classes into finer subtypes, such as CGRP-α and CGRP-η nociceptors, each with distinct receptor expression, central projections, and response thresholds [40]. Moreover, Qi et al. expanded this framework by providing new insights into transcriptional diversity and functional specialization of sensory neurons [41]. Although these neuron classes are largely conserved across species, key differences in the expression of signaling molecules (e.g., TAFA4) and ion channels reflect divergent adaptations in nociceptive and pruriceptive pathways [42]. Collectively, these insights support a population coding mechanism in which the combinatorial activation of highly specialized subtypes—including NF neurons, LTMRs, proprioceptors, C-LTMRs, and PEP/NP nociceptors—encodes the full spectrum of somatosensory stimuli.

Most itch-transmitting neurons are NP neurons, including the NP1, NP2, and NP3 subclusters. NP1 neurons, identified by the marker Mas-related G protein-coupled receptor member D (MrgprD), are polymodal sensory neurons involved in touch, pain, and itch. MrgprD^+^ neurons mediate pain through the release of glutamate, while itch signaling is facilitated by the synergistic action of glutamate and NMB [43, 44]. NP2 neurons, characterized by the expression of Mas-related G protein-coupled receptor A3 (MrgprA3), mediate histamine-independent itch. They are selectively activated by nonhistaminergic pruritogens, including chloroquine and bovine adrenal medulla peptide 8–22 (BAM8-22) [20, 21]. NP3, marked by somatostatin (SST), mediates itch via disinhibition of spinal gastrin-releasing peptide receptor (GRPR)^+^ neurons [45–48]. Recent studies have shown that mechanically induced itch is mediated by Toll-like receptor 5 (TLR5).^+^ Aβ fibers, which play a key role in alloknesis under pathological conditions [49]

### Spinal mechanisms of itch

**Fig. 1:**
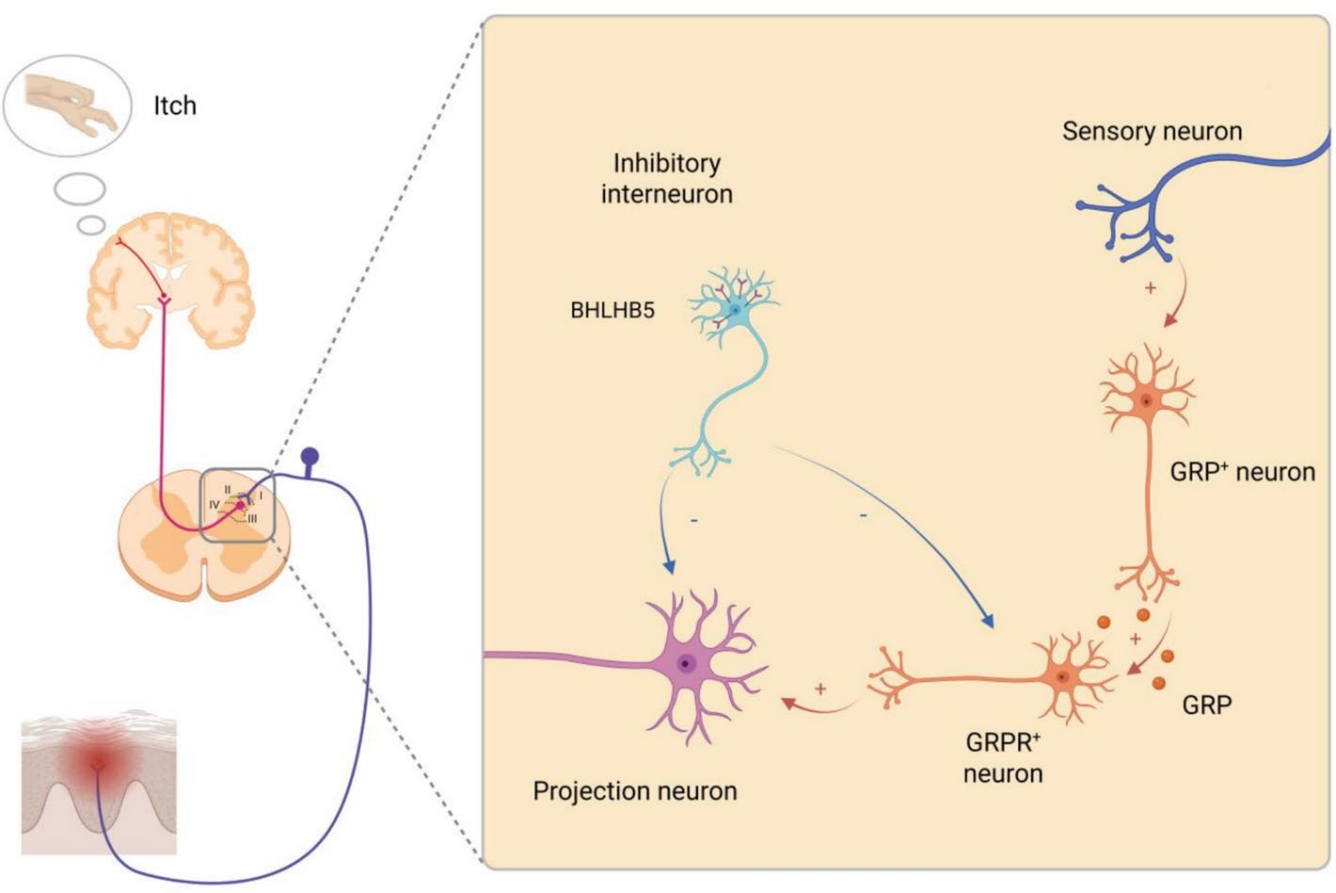
Spinal mechanisms of itch. After pruritogens stimulate the skin, itch-sensitive primary afferents (pruriceptors) project to the superficial dorsal horn (laminae I–II). Within the dorsal horn, GRP^+^ excitatory interneurons release gastrin-releasing peptide (GRP) to activate GRPR^+^ excitatory relay interneurons, which in turn drive projection neurons (often NK1R^+^). This excitatory pathway is gated by GABAergic and glycinergic inhibitory interneurons, including Bhlhb5^+^ cells. Projection neurons ascend via the spinoparabrachial pathway to the parabrachial nucleus and via the spinothalamic tract to thalamic and cortical targets, where itch perception emerges. The notations “ + ” and “–” denote excitatory and inhibitory effects, respectively. This figure was created with BioRender (https://www.biorender.com/)

Primary pruriceptive afferents terminate predominantly in laminae I–II of the spinal dorsal horn (SDH), where itch signals are processed by networks of excitatory and inhibitory interneurons before reaching projection neurons. A core excitatory pathway involves gastrin-releasing peptide (GRP)^+^ interneurons that activate gastrin-releasing peptide receptor (GRPR)^+^ neurons, which then drive NK-1R-expressing projection neurons ascending via spinothalamic and spinoparabrachial tracts [23, 115–117]. This pathway is under inhibitory control by glycinergic/GABAergic interneurons, including Bhlhb5^+^ (B5-I) cells that release dynorphin to suppress pruriceptive transmission [118, 119]. A schematic overview of these spinal components and their connectivity is provided in Fig. 1.

Beyond this basic relay, subsequent studies have shown that spinal itch circuits are highly modular. Multiple excitatory populations (e.g., GRPR^+^, Ucn3^+^, and NPY1R^+^ interneurons) and overlapping inhibitory modules (glycinergic/GABAergic, dynorphinergic, and galaninergic) shape gain and timing [120–122]. Crosstalk with nociceptive pathways provides additional control, with μ-opioid signaling disinhibiting itch and κ-opioid signaling suppressing it, consistent with the antipruritic effects of κ-agonists. At the transmitter level, glutamate via AMPA/kainate receptors mediates primary afferent input, while neuropeptides such as GRP act as modulators [23, 115, 116]. Mechanical itch engages partly distinct elements: under pathological conditions, TLR5^+^ Aβ afferents recruit urocortin 3-positive (Ucn3^+^) excitatory neurons, while an NPY-lineage inhibitory “gate” normally prevents touch-to-itch conversion [49, 120, 122]).

Projection neurons are themselves heterogeneous. While many express NK-1R, distinct subsets within spinothalamic and spinoparabrachial tracts likely encode the intensity, quality, and affective dimensions of pruritus [121, 123]. Descending monoaminergic modulation further sculpts SDH processing and helps explain differences between acute and chronic itch. In addition, chronic itch involves central sensitization, with astrocyte- and microglia-derived signals potentiating GRPR-pathway excitability and sustaining pruriceptive gain (as discussed in detail in Sect. 3) [121, 122]. 

## Neuroinflammatory regulation of sensory neurons in itch

### Itch-related immune receptors in pruriceptors

Pruriceptive neurons express various immune receptors and can be activated by corresponding immune mediators under itch conditions, constituting a key mechanism of peripheral sensitization. A comprehensive understanding of these immune receptors is essential for the development of targeted therapies to alleviate intractable itch. The following section provides a detailed description of pruriceptive immune receptors in itchy sensory neurons and their roles in itch diseases, as summarized in Table 1.

**Table 1:** The immune receptors in pruriceptors: preclinical and clinical evidence

Receptor group	Receptor subtype	Population	Ligand	Ligand source	Related disease	
					Preclinical evidence	Clinical evidence
Histamine receptor	H1R	NP2 and NP3 DRG sensory neurons	Histamine	Mast cell, basophils	Chronic spontaneous urticaria in mice [50, 51]	Chronic spontaneous urticaria; mosquito bite allergy [52–54]
	H3R	DRG sensory neurons			Inhibit itch in rats and mice and H3R knockout mouse model [50, 55]	/
	H4R	NP2 and NP3 DRG sensory neurons			Allergic contact Dermatitis; atopic dermatitis in mice [56, 57]	Allergic contact Dermatitis; atopic dermatitis [57, 58]
Interleukin receptor	IL-4Rα	NP1, NP2, NP3, DRG	IL-4	Th2	Atopic dermatitis, chronic itch in mice [30, 59, 60]	Atopic dermatitis, chronic itch [61, 62]
	IL-31Rα	Itch-specific sensory neurons with NP3 and TRPV1, DRG	IL-31	Th2, granulocytes	Atopic dermatitis, chronic itch in mice [63–65]	Atopic dermatitis, chronic itch,
						spontaneous urticaria, psoriasis [45, 66–70]
	TSLPR	Epithelial cells, dendritic cells, mast cells, and fibroblasts	TSLP	Keratinocytes	Atopic dermatitis in mice in mouse model [71, 72]	Atopic dermatitis, psoriasis, chronic spontaneous urticaria, and dermatitis herpetiformis [71, 73–75]
	IL-33R/ST2	Neurons sensitive to chloroquine and histamine	IL-33	Epithelial cells	Allergic contact dermatitis in mouse model (poison ivy contact allergy) [76–78]	Allergic dermatitis [76, 79, 80]
	IL-3R	Sensory neurons	Il-3	GD3	Allergic contact dermatitis in Tcrd^GDL^ and iTcrd^DTA^ mouse model [81, 82]	Allergic contact dermatitis [81]
	OSMR	Itch-selective Nppb neurons	Oncostatin M	Dermal T cells, monocytes	Delayed-onset itch, chronic itch in OSMR knockout mouse model [83]	Prurigo nodularis, atopic dermatitis, psoriasis [84]
Chemokine receptor	CCR2	Sensory neurons	CCL2	Keratinocytes, endothelial cells, macrophages, fibroblasts, dendritic cells, DRG	Allergic contact dermatitis in allergic contact dermatitis mouse model [85–87]	/
	CXCR3		CXCL10			
	CXCR4		CXCL12			
TLRs	TLR3	Sensory nerves within DRG, intracellular vesicles	Poly I:C	Internalized pathogens	Histamine-dependent and histamine-independent itch in TLR3 knockout mouse model [88, 89]	/
	TLR4	Sensory nerves, microglia, astrocyte, plasma membrane	LPS	Extracellular PAMPs	Chronic itch, histamine-independent itch, and atopic dermatitis in TLR4 knockout mouse model [90, 91]	/
	TLR7	Sensory nerves within DRG, intracellular vesicles	Imiquimod, poly I:C	Internalized pathogens	Histamine-independent itch in TLR7 knockout mouse model [89, 92]	/
PAR	PAR2	DRG	Exogenous or endogenous proteases	Mast cells, plants	Histamine-independent itch in rats and atopic dermatitis in mouse and rat model [93–96]	Atopic dermatitis [93, 97]
Others	FcεR	Soma and fibers of MrgprA3^+^, trigeminal neurons	IgE-IC	Antigen	Itch, ocular itch in allergic conjunctivitis mouse and rat model [98, 99]	Allergic conjunctivitis [100, 101]
	MRGPRX2	DRG	SP	Cutaneous sensory nerve endings, mast cells	Chronic urticaria in mice and cells (mast cells degranulation) [102, 103]	Chronic urticaria [102, 104]
	Endothelin receptors	DRG, Keratinocytes and endothelial cells	Endothelin-1	Spinal cord and sensory neurons, keratinocytes, endothelial cells	Atopic dermatitis in atopic dermatitis mouse model and human HaCaT keratinocyte cell line [105]	Atopic dermatitis, psoriasis, prurigo nodularis [106, 107]
	HTR	DRG	5-HT	Mast cells	Pain and itch in spontaneous scratches mice model, atopic dermatitis in atopic dermatitis mouse and rat model [108–110]	Pruritus, uremic pruritus, and cholestatic pruritus [111–114]

#### Histamine receptors

Histamine, a classical pruritogen, has been extensively studied for its role in itch, which is broadly categorized into histamine-dependent and histamine-independent types. Mast cells and basophils store large amounts of histamine in their granules and serve as the major reservoir of histamine in the human body. During the development of itch, histamine is predominantly released from mast cells or basophils upon degranulation and directly activates sensory neurons via histamine receptors, inducing pruritus [47, 124, 125]. Four primary histamine receptors (H1R, H2R, H3R, and H4R) have been identified. In mice and guinea pig DRG, sensory neurons mainly express H1R and H4R, primarily localized in nonpeptidergic, itch-specific NP2 and NP3 neuronal subpopulations [38, 126–128]. Similarly, studies on human tissues have detected H1R expression in sensory neurons, suggesting conserved mechanisms of histamine receptor-mediated itch between mice and humans [129]. Therefore, H1R antagonists and mast cell stabilizers are the most commonly used types of clinical medications for relieving itch. Moreover, cadaverine, a microbiome-derived metabolite, induced itch in an H4R- and transient receptor potential vanilloid 1 (TRPV1)-dependent manner in mice [130]. Additionally, H3R is also detected in mice DRG sensory neurons, where it functions as an inhibitory receptor for itch and performs reverse effects compared with H1R and H4R [50, 55]. Furthermore, histamine induces calcium influx in sensory neurons through the phospholipase A2 (PLA2)/lipoxygenase/TRPV1 signaling pathway, as demonstrated in rat models. TRPV1 has been identified as the primary downstream ion channel coupled with H1R, mediating histamine-dependent itch [22, 131, 132]. Interestingly, small ubiquitin-like modifier modification (SUMOylation) of TRPV1 has been shown to suppress itch by inhibiting the interaction between TRPV1 and H1R in mouse model [133]. Antihistamines remain widely used to treat itch and are often effective.

Although the histaminergic pathway plays a role in certain types of itch, antagonism of H1R or H2R is ineffective in most chronic itch conditions, with the exception of chronic urticaria [56]. The perceived antipruritic benefit of first-generation antihistamines is largely attributable to sedative effects mediated by central nervous system activity, whereas second-generation agents cause less sedation but remain clinically useful mainly for urticaria and mastocytosis [134]. By contrast, H4R antagonists have shown antipruritic effects in mouse models, suggesting a potential target for future therapies [56]. Overall, H1R/H2R antihistamines demonstrate limited therapeutic value for chronic itch beyond urticaria.

#### Interleukin receptors

##### IL-4Rα

Hallmark Th2 cytokines interleukin-4 (IL-4) and interleukin-13 (IL-13) signal through a receptor complex composed of IL-4Rα and IL-13Rα1, triggering the Janus kinase (JAK)/signal transducer and activator of transcription (STAT) pathway [135]. These cytokines are expressed and released by hematopoietic cells, including eosinophils, basophils, and mast cells [136]. Recent evidence confirms the presence of IL-4Rα and IL-13Rα1 in the DRG of both mice and humans, and shows that IL-4 and IL-13 can directly activate sensory neurons [30, 59, 60]. Analysis of a canonical single-cell RNA-seq dataset of mouse neuron revealed that small-diameter neuron subtypes associated with itch—NP1, NP2, and NP3—exhibited higher expression of IL-4Rα and IL-13Rα1 than nociceptive (PEP1 and PEP2) and mechanoreceptive populations, suggesting that IL-4Rα^+^ and IL-13Rα1^+^ neurons are predominantly pruriceptive [38]. Furthermore, neurons from *Trpv1*^*−/−*^ mice showed markedly diminished responses to IL-4 and IL-13, indicating that these cytokines activate sensory neurons via a TRPV1-dependent calcium influx mechanism [137, 138]. This highlights the critical role of TRPV1 in mediating IL-4- and IL-13-induced neuronal activation and suggests its involvement in the downstream mechanisms of itch signaling. Moreover, conditional knockout of *Il4ra* in Nav1.8^+^ sodium channels (Nav1.8)^+^ nociceptors and inhibition of downstream JAK1 signaling significantly alleviated itch in an AD model [30, 139].

IL-4 and IL-13 have been shown in vitro to downregulate key differentiation markers of keratinocytes—including filaggrin, loricrin, and involucrin—indicating their potential contribution to epidermal barrier impairment [140–142]. Furthermore, individuals with atopic dermatitis exhibited increased circulating IL-4 and IL-13 relative to healthy subjects, although these levels did not correlate with pruritus intensity. Among those with psoriasis, IL-4 concentrations exceeded those observed in control groups, whereas IL-13 levels were comparatively reduced [80]. This mechanistic insight has driven the development and clinical evaluation of multiple biologics aimed at modulating IL-4/IL-13 activity. Therapeutic approaches include blocking cytokine-receptor binding (e.g., dupilumab, tralokinumab, or lebrikizumab) or targeting downstream signaling cascades via JAK inhibitors such as tofacitinib, baricitinib, delgocitinib, upadacitinib, and ruxolitinib [143]. Among these, dupilumab has the strongest clinical evidence supporting both efficacy and long-term safety in chronic itch conditions. IL-13-targeting antibodies such as tralokinumab and lebrikizumab show promising results but remain under active investigation. JAK inhibitors have also demonstrated antipruritic efficacy across multiple trials; however, safety concerns necessitate cautious use and ongoing clinical monitoring [144].

##### IL-31Rα

Interleukin-31 (IL-31), a cytokine known to induce itch, is predominantly produced by Th2 lymphocytes and, to a lesser extent, by granulocytes [145]. Its main receptor subunit, IL-31Rα, associates with oncostatin M-specific receptor β (OSMRβ) to form a functional signaling complex. Expression of IL-31Rα is primarily found in NP3-type pruriceptive sensory neurons that also express TRPV1, and it has been identified in dorsal root ganglia of both humans and mice, as well as in keratinocytes, innate immune populations, eosinophils, T lymphocytes, and peripheral cutaneous nerve fibers [80, 145, 146].

IL-31-evoked itch is markedly reduced in mice lacking TRPV1 or TRPA1, but remains unaffected in c-kit or PAR-2 knockout models. In primary sensory neuron cultures, IL-31 triggers intracellular Ca^2+^ release and phosphorylation of extracellular signal-regulated kinase (ERK)1/2; pharmacological blockade of these pathways suppresses IL-31 signaling in vitro and attenuates IL-31-induced scratching in vivo [146, 147]. In addition to directly activating sensory neurons and inducing itch, IL-31 amplifies itch responses to various pruritogens in a STAT3-dependent manner. In an AD mouse model, the deletion of *Il31ra* or *Stat3* in Nav1.8^+^ nociceptors significantly alleviated pathological itch. These findings suggest that IL-31 activates a neuroinflammatory cascade in pruriceptors and mediates itch through the IL-31Rα-ERK/STAT3 signaling pathway [63]. Furthermore, activation of IL-31Rα^+^ neurons facilitates skin inflammation in AD mouse model through neuropeptide-mediated neuroinflammation [148, 149]. Inhibition of this pathway has shown potential to alleviate both skin inflammation and itch in inflammatory skin diseases. Individuals with AD exhibit elevated IL-31 levels in plasma compared with healthy controls, with a stronger increase noted in pediatric cases relative to adults [45, 66]. Additionally, IL-31Rα expression is upregulated in AD skin compared with nonlesional skin [67]. These findings underscore the involvement of IL-31 signaling in both the development of AD and the associated pruritus. Elevated IL-31 concentrations have also been identified in spontaneous urticaria, mastocytosis, and psoriasis, although no clear link to itch intensity has been established in psoriasis [68–70, 150]. In contrast, prurigo nodularis exhibits a robust correlation between itch severity and dermal IL-31/IL-31RA/OSMRβ expression [151]. Specific therapies targeting IL-31 or its receptor (e.g., nemolizumab) have shown significant anti-itch efficacy in clinical trials, particularly for AD and other pruritic conditions [147, 152–154].

##### Thymic stromal lymphopoietin receptor

Thymic stromal lymphopoietin (TSLP), produced by keratinocytes in response to stimuli such as activation of protease-activated receptor-2 (PAR-2), is a key epithelial cytokine driving Th2-type inflammation [155]. TSLP is absent in healthy skin but highly expressed in the lesional skin of patients with AD—localized in keratinocytes, epithelial cells, dendritic cells, mast cells, and fibroblasts [73]. Its receptor, comprising the shared subunit IL-7Rα and the TSLP-specific subunit TSLPR, is expressed in sensory neurons of both mice and humans, with both components localized to a discrete nociceptor population distinct from histamine- and chloroquine-responsive neurons. TSLP directly induces itch by activating sensory neurons via the TSLP receptor and signals downstream in a TRPA1-dependent manner [156].

TSLP has a well-established link to the pathophysiology of AD [157]. Its expression is markedly elevated in lesional, especially apical epidermal, regions of the skin of patients with AD, while nonlesional sites from the same patients lack this elevation [73]. The degree of overexpression aligns with clinical disease severity scores such as the SCORing Atopic Dermatitis (SCORAD) index [74]. Keratinocyte-derived TSLP contributes to pruritus by activating peripheral sensory neurons and drives inflammatory responses via dendritic cell recruitment and migration [158]. In the context of AD, TSLP also upregulates nuclear IL-33 in keratinocytes by triggering the mitogen-activated protein kinase (MAPK) cascade (extracellular signal-regulated kinase (ERK)/c-Jun N-terminal kinase), thereby compromising the epidermal barrier [159]. Tezepelumab, an IgG2 monoclonal antibody against TSLP, is currently being tested in multiple trials, though its standalone therapeutic benefit for AD is yet to be validated [160]. Beyond atopic dermatitis, TSLP has been investigated in other pruritic dermatoses such as psoriasis, chronic urticaria, and dermatitis herpetiformis, where elevated levels are often linked to pruritus severity [71].

##### IL-33R/ST2

Interleukin-33 (IL-33), a member of the IL-1 cytokine family, promotes production of Th2 cytokines by activating a range of immune cell types [161]. IL-33 is normally expressed in intact skin and can be secreted by epithelial cells, where it acts to trigger and enhance Th2-driven immune responses in atopic dermatitis [28]. Keratinocyte-derived IL-33 has been implicated as a key contributor to itch in allergic contact dermatitis (ACD) triggered by agents such as urushiol and oxazolone [76]. Its receptor, comprising the IL-33-specific ST2 subunit and the accessory chain IL-1RAcP, is expressed in neurons responsive to histamine and chloroquine. Although IL-33 alone induces only modest Ca^2+^ influx and no itch in naïve animals, both IL-33 and ST2 are essential for sustaining itch, particularly in urushiol-associated pruritus. IL-33-induced neuronal activation depends on ST2 signaling and involves TRPV1 and TRPA1 channel engagement [76, 77]. Elevated IL-33 levels have been detected in both the skin and circulation of individuals with atopic dermatitis and idiopathic pruritus, underscoring its pathogenic role in these conditions [78, 79]. In contrast, patients with psoriasis exhibit reduced IL-33 levels in plasma compared with healthy subjects [80].

##### IL-3R

Research into the function of interleukin-3 (IL-3) and its corresponding receptor in pruritus is still in its early stages. Cameron et al. identified an epidermal γδ T cell population, GD3 cells, whose characteristics remain incompletely defined. These cells release IL-3, a key cytokine that contributes to allergic itch and initiates type 2 immune responses [81, 82]. On a mechanistic level, IL-3 interacts with IL-3Rα-expressing sensory neurons through a Janus kinase 2 (JAK2)-dependent pathway, reducing the allergen response threshold without directly triggering itch. This γδ T cell–IL-3 axis also engages STAT5 signaling, enhancing neuropeptide expression and initiating allergic inflammation [82]. Collectively, these findings suggest the existence of an intrinsic immune modulator acting upstream of sensory neurons to fine-tune their reactivity during initial allergen encounters. Such a mechanism may underlie variability in allergic sensitivity and provide novel targets for therapeutic intervention in allergic diseases.

##### Oncostatin M receptor

Oncostatin M (OSM) is significantly overexpressed in pruritic dermatoses (e.g., psoriasis and AD), primarily by dermal T cells and monocytes. Its receptor OSMR and co-receptor gp130 are specifically expressed in itch-selective natriuretic polypeptide B (NPPB) neurons [83]. Unlike direct neuronal activators, OSM induces itch via dual neural sensitization: short-term enhancement of responses to pruritogens such as histamine and long-term increase of neuronal excitability (converting action potentials from phasic to tonic). Intradermal OSM alone induces delayed-onset itch (≥ 30 min) and potentiates histamine/leukotriene-induced itch [84]. Sensory neuron-specific OSMR knockout or gp130 inhibitor SC144 treatment alleviated scratching and skin inflammation in mouse itch models, establishing OSM as a core neuroimmune modulator and novel therapeutic target in itch [84]. Suehiro et al. identified a dual OSM itch regulation mechanism: OSM (elevated in AD/psoriatic lesions; undetectable in most healthy skin; released by IL-4/GM-CSF-activated monocytes) systemically downregulates DRG neuronal IL-31Rα (*p* < 0.01) and upregulates IL-4 Rα/IL-13Rα1 [84]. In vitro, OSM decreases keratinocyte IL-31Rα but increases OSMR. OSM pretreatment (12 h) suppressed IL-31-induced scratching in mice (213.8 versus 321.7 bouts, *p* < 0.05) without affecting histamine/5-HT itch. Thus, OSM negatively regulates IL-31-related itch via monocyte-keratinocyte feedback and neuronal IL31Rα suppression, while enhancing IL-4/IL-13 signaling, offering a novel therapeutic approach pending clinical validation.

The anti-OSMRβ monoclonal antibody vixarelimab improved both pruritus and skin lesions in patients with moderate-to-severe prurigo nodularis. By week 8, vixarelimab treatment produced a greater reduction in itch intensity and a higher proportion of patients achieving clear or almost clear skin compared with placebo. Adverse events were comparable between groups, with no serious events reported. As the first agent targeting OSMRβ, vixarelimab simultaneously inhibits IL-31-mediated pruritus and OSM-driven inflammation and fibrosis, demonstrating potential as a targeted therapy for prurigo nodularis [162].

#### Toll-like receptors

Traditionally, innate immunity has been understood as relying on Toll-like receptors (TLRs) expressed by epithelial and immune cells to recognize pathogen-associated molecular patterns (PAMPs), including components of bacterial cell walls [163–167]. Among the 12 known mouse TLRs, TLR1/2, TLR2/6, TLR4, TLR5, and TLR11 are positioned on the plasma membrane to sense extracellular PAMPs, whereas TLR3, TLR7, TLR8, and TLR9 reside within intracellular vesicles to detect nucleic acid-based PAMPs from internalized pathogens [168, 169]. TLR3 and TLR7 are selectively expressed in pruriceptive neurons of the DRG, where their activation by ligands such as poly I:C and imiquimod enhances neuronal excitability and promotes itch. Unlike their canonical intracellular roles, TLR3 and TLR7 appear to act at the plasma membrane in sensory neurons; knockout of either gene resulted in attenuated scratching behavior in itch models, with TLR7 specifically mediating histamine-independent itch and TLR3 contributing to both histamine-dependent and histamine-independent pathways [88, 89, 92]. A recent study identified TLR5 expression in a distinct subset of Aβ large-diameter, thickly myelinated nerve fibers. These fibers mediate mechanical itch alloknesis through synaptic connections with spinal Ucn3^+^ neurons [49, 170]. While TLR-mediated itch mechanisms remain incompletely elucidated, cell-type-specific genetic approaches could clarify whether they activate sensory neurons directly, interact with peripheral nonneuronal cells, or modulate itch circuits.

Direct TLR stimulation shows therapeutic benefit: Topical 5% *Veronica filiformis* lysate (TLR2 ligand) improved AD symptoms; γPGA (TLR4 ligand) reduced AD symptoms, basophils, and Th2 cytokines while upregulating Th1 cytokines via TLR4/DC/IL-12 in mice; *Tenebrio molitor* trypsin hydrolysate alleviated AD symptoms in a mouse model via TLR2/MyD88 inhibition; TLR9 agonists (e.g., CpG ODN) showed efficacy in canine/mouse AD, cutaneous allergen immunization, and Th2 diseases [171–178]. Conversely, conjugated linoleic acid inhibits TLR4/MyD88/NF-κB to reduce AD symptoms, while TLR8 modulation via *Salmonella*-delivered miRNA and herbal agents (e.g., calycosin and osthole) targeting TLR pathways improve AD characteristics [179–182]. Similarly, given the pivotal role of TLRs in AD pathogenesis, they represent promising AD therapeutic targets. Existing AD treatments partially function through TLR modulation: topical vitamin D analogs promote TLR2-mediated cathelicidin production in keratinocytes to alleviate infection and itch; tacrolimus reduces TLR1 expression and restores TLR2 function in AD lesions; cyclosporin enhances TLR2-responsive innate immunity in keratinocytes; and H4R antagonists suppress CCL17/22 production while potentiating TLR2 signaling to mitigate pruritus [90, 91, 179, 183–185]. Phototherapy immunosuppression depends on TLR3 and TLR4 [186, 187].

#### Chemokine receptors

Three key chemokine receptors expressed by sensory neurons, viz. C–C motif chemokine receptor 2 (CCR2), C–X–C motif chemokine receptor 3 (CXCR3), and C–X–C motif chemokine receptor 4 (CXCR4), are critical mediators of itch. In a mouse ACD model, these receptors were significantly upregulated in sensory neurons, particularly nociceptors. ACD also heightened the excitability of sensory neurons innervating affected skin in response to C–C motif chemokine ligand 2 (CCL2) (CCR2 ligand) or C–X–C motif chemokine ligand 10 (CXCL10) (CXCR3 ligand). Notably, CXCL10/CXCR3 signaling mediates allergic itch without contributing to inflammatory pain during skin inflammation, whereas blocking CCR2 or CXCR4 alleviates both itch and pain associated with ACD [85–87]. Neutrophils contribute to itch by producing CXCL10, which activates CXCR3 on sensory neurons. In a mouse AD model, blocking the CXCL10–CXCR3 axis reduced scratching behavior, highlighting a role for neutrophils as initiators of itch and suggesting CXCR3 as a potential therapeutic target for chronic itch [188]. Mechanistically, Cl^−^ channels act as downstream effectors of CXCL10, mediating excitatory and pruritic behavioral responses. Thus, targeting Cl^−^ channels represent a promising therapeutic approach for managing allergic itch driven by CXCL10/CXCR3 signaling [189]. Further research is required to elucidate the downstream signaling pathways of CCR2 and CXCR4 to better understand their distinct contributions to pain and itch.

#### Adaptive immune receptors

The classical immune receptor pathway involves immunoglobulin E (IgE)-mediated itch, where subsequent allergen exposure triggers allergen-specific IgE crosslinking on mast cells and basophils. This interaction releases histamine and other vasoactive mediators (e.g., substance P, bradykinin, prostaglandins, and leukotrienes) [190]. Histamine binds receptors on unmyelinated C fibers, transducing and transmitting itch signals [191]. Recent studies have revealed that immune complexes can directly activate sensory neurons expressing adaptive immune receptors in mice and in vitro, such as the Immunoglobulin G (IgG) receptor Fc gamma receptor (FcγR) and the IgE receptor Fc epsilon receptor (FcεR) [192]. Notably, these receptors exhibit distinct sensory properties: FcγR^+^ neurons mediate joint pain in antigen-induced arthritis (AIA), while FcεR^+^ neurons mediate ocular itch in ACJ [98, 193]. The high-affinity IgE receptor FcεRI is composed of four subunits: an α-chain (FcεRIα) responsible for IgE binding, a β-chain (FcεRIβ), and two γ-chains (FcεRIγ) that mediate signal transduction [194, 195]. FcεRIα is present in both the cell bodies and axons of MrgprA3^+^ sensory neurons, which respond directly to IgE-immune complexes (IgE-IC). Allergen sensitization enhances FcεRI expression on these neurons, thereby potentiating their responsiveness to immune complex stimulation. IgE-IC can directly induce ocular itch through FcεRIα-dependent activation of trigeminal neurons. In a mouse ACJ model, FcεRIα expression was significantly upregulated in CGRP^+^ pruriceptors, and knockdown of FcεRIα in sensory neurons substantially alleviated ocular itch associated with ACJ [99]. Additionally, previous studies in rats have shown that neuronal FcγRI functionally couples with transient receptor potential canonical 3 to regulate the excitability of DRG neurons [196]. Further research is needed to elucidate the detailed neural signaling and ion channel responses to IgE-IC.

#### Protease-activated receptors

Protease-activated receptors (PARs), members of the G protein-coupled receptor (GPCR) family, are widely expressed in various tissues, including the DRG [93, 94]. PARs primarily mediate nonhistaminergic itch. Proteases, whether internal or external, trigger receptor activation by cleaving the N-terminal domain, generating a tethered ligand that subsequently initiates signaling through the same receptor. Endogenous serine proteases such as kallikreins (KLK5 and KLK14) activate PAR2 in vitro [197]. Cathepsin S induces human itch and activates human PAR2/PAR4 in vitro; correspondingly, cathepsin S-induced itch is attenuated by cathepsin S inhibitors, PAR2 antagonists, and TRPV1 knockout [95, 97]. Mast cell-derived tryptase under pruritic states can also stimulate PAR2 activity [156]. The role of PAR2 in pruritus is further supported by the antagonist PZ-235 attenuation of itch induced by house dust mite and mast cell degranulation [96].

Exogenous proteases can induce itch by activating protease-activated receptors. In mice and human skin, *Staphylococcus aureus* secretes the V8 serine protease that directly activates PAR1 on pruriceptor neurons, driving itch and scratch-induced barrier damage; pharmacologic or genetic interference with V8-PAR1 signaling reduces itch behaviors and tissue injury [198]. Cowhage’s cysteine protease mucunain evokes nonhistaminergic itch via PAR2/PAR4 activation [199]. Similarly, plant-derived cysteine proteases such as bromelain, ficin, and papain can activate PAR2/PAR4, supporting a broader role for PAR signaling in protease-evoked itch [97].

#### Serotonin receptors

Although serotonin (5-hydroxytryptamine, 5-HT) is a classical neurotransmitter in the CNS, it can also be released from mast cells in the periphery independently of histamine [200, 201]. Its pruritogenic effects are mediated through specific serotonin receptors (HTRs) expressed on sensory neurons. When applied to human or mouse skin at high doses (> 1 mM), 5-HT elicited both pain and itch sensation [111, 202, 203], whereas lower doses of 5-HT (100 μM) only cause itch [108]. There are 14 known 5-HT receptors, grouped into seven classes. Several 5-HT receptors have been detected in DRG neurons [38, 108, 204]. Antagonists to HTR1 and 2 and HTR3 have been shown to reduce 5-HT-mediated itch [109–111]. Disruption of the *Htr7* gene entirely eliminated scratching behavior triggered by low-dose serotonin and significantly diminished pruritus in a mouse AD model [108]. Unlike other serotonin receptors, which are G protein-coupled, HTR3 functions as a ligand-gated ion channel [109]. HTR7, a Gs-protein-coupled receptor, initiates neuronal excitation by activating TRPA1 via the adenylyl cyclase–cyclic adenosine monophosphate (cAMP) signaling pathway in mice [108]. Additional evidence suggests that both phospholipase C beta 3 (PLCβ3) and the TRPV4 channel are essential for serotonin-induced itch in both mouse and human, likely acting downstream of another subtype of 5-HT receptor [22, 205].

#### Leukotriene receptors

Leukotrienes are lipid mediators involved in multiple allergic diseases. While their role in asthma and allergic rhinitis is well established, their contribution to atopic dermatitis (AD) is less clearly defined [206–211]. Elevated leukotriene levels have been observed in AD and may contribute to pathogenesis by increasing vascular permeability, recruiting inflammatory cells, and promoting chronic tissue changes in mouse models [212]. Although leukotriene receptor antagonists such as montelukast are mainly prescribed for asthma, limited off-label benefit has been reported in AD, particularly in patients with overlapping allergic conditions. Consistently, urinary leukotriene E_4_ (LTE_4_) levels are elevated in patients with AD and correlate with disease severity [213].

Recent work has clarified a neuronal mechanism linking leukotrienes to itch. Cysteinyl leukotriene receptor 2 (CysLT_2_R), expressed in sensory neurons, mediates leukotriene C_4_ (LTC_4_)-induced itch independently of mast cells [214]. In mouse models of AD, basophils recruited upon allergen exposure release LTC_4_, which activates neuronal CysLT_2_R to drive acute itch flares, suggesting a basophil–neuronal axis in inflammatory itch [215]. These findings highlight CysLT_2_R signaling as a potential therapeutic target for chronic and allergic itch.

### Itch-related immune mediators from sensory neurons

**Fig. 2:**
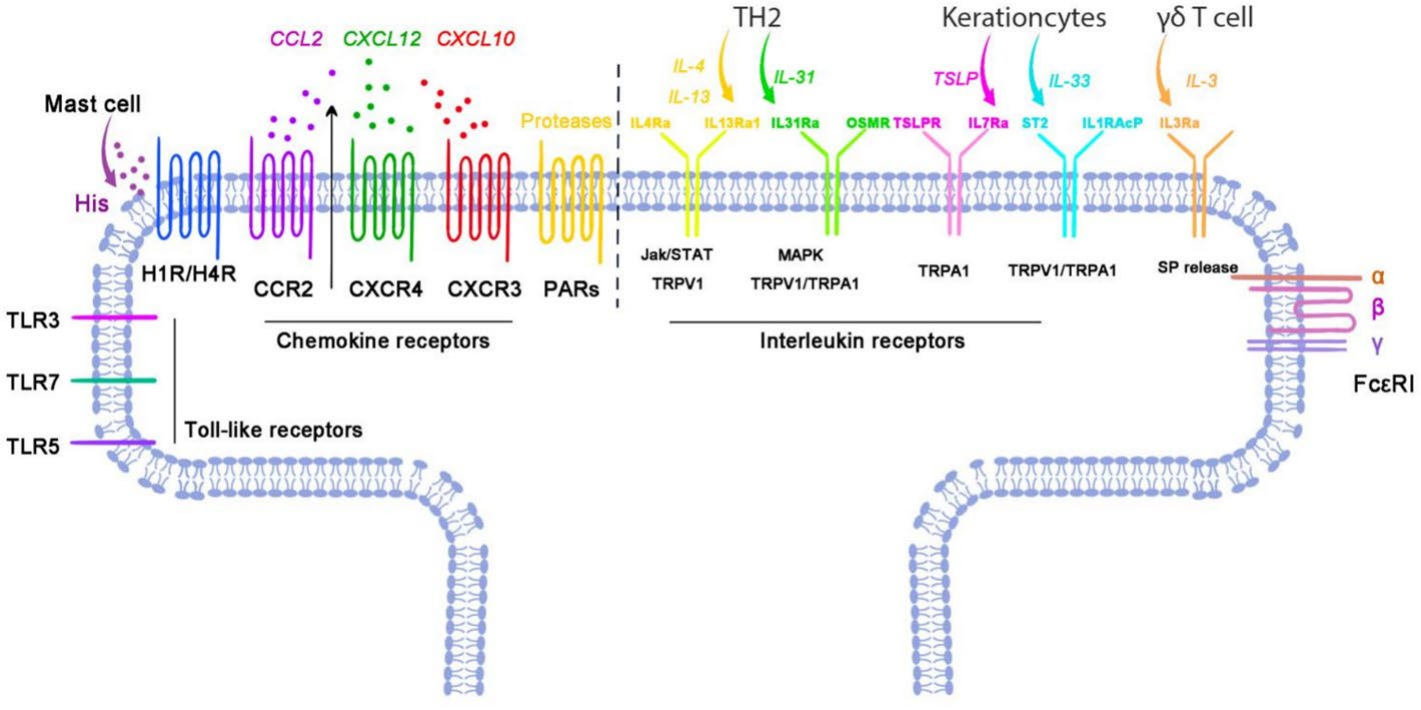
Neuro-inflammatory regulation of sensory neurons under itch conditions. Receptors on sensory neurons are grouped into G-protein-coupled receptors (e.g., histamine H1/H4R, Mrgprs, PARs, an.d chemokine CCR2/CXCR4/CXCR3), cytokine receptors (e.g., IL-4Rα/IL-13Rα1, IL-31Rα/OSMRβ, TSLPR/IL-7Rα, ST2/IL-1RAcP, and IL-3Rα), ion channels (e.g., TRPV1 and TRPA1), and immune-recognition/other receptors (e.g., TLR3/5/7 and FcεRI complex). Arrows indicate representative cell-to-neuron pathways from Th2 cells (IL-4/IL-13, and IL-31), mast cells (histamine and proteases), keratinocytes (TSLP and IL-33), and γδ T cells (IL-3). Together, these inputs directly excite or sensitize pruriceptors and couple to downstream TRPV1/TRPA1 and JAK–STAT/MAPK signaling, initiating itch. The diagram is illustrative rather than exhaustive; abbreviations are defined in the list of abbreviations. The figure was created with Adobe Photoshop

The schematic in Fig. 2 summarizes how immune cues drive pruriceptor (primary afferent) activation in the skin and dorsal root ganglion (DRG).

With the neuron-expressed immune receptors summarized in Fig. 2, we next describe how neuron-derived mediators (e.g., glutamate, neuropeptides, and cytokines) engage immune and cutaneous cells to modulate pruriception.

#### Substance P

Substance P (SP), a neuropeptide, primarily exerts its effects via neurokinin receptors NK1, NK2, and NK3, which are members of the GPCR family, with NK1 displaying the greatest binding affinity. SP is predominantly secreted by skin-resident sensory neurons, though keratinocytes and other cell types can also contribute to its release [216, 217]. NK1 receptors are widely distributed across various cell types linked to pruritus signaling, including immune cells, fibroblasts, endothelial cells, and cutaneous sensory terminals. Recent findings have highlighted MrgprX2 as a receptor for SP that facilitates mast cell degranulation, triggering the release of histamine and tryptase in an SP-dependent fashion [102]. Elevated MrgprX2 levels on skin mast cells have been noted in individuals with chronic urticaria [104]. This reciprocal signaling further intensifies the itch sensation, as mast cell-derived mediators can promote additional SP release from neurons.

This neuroimmune mechanism was elegantly demonstrated in a model of allergic skin inflammation induced by house dust mites (HDMs). HDMs activate TRPV1^+^Tac1^+^ nociceptors, leading to SP release and subsequent mast cell degranulation via MRGPRB2 (the mouse ortholog of MrgprX2). This SP-mediated signaling between neurons and mast cells is essential for the development of type 2 skin inflammation and highlights MRGPRB2 as a critical receptor in neuroimmune communication. The functional cluster formed by nociceptors and mast cells thus operates as a key sensorimotor unit that initiates allergic skin reactions upon environmental allergen exposure.

#### Endothelin-1

Endothelin-1 (ET-1) is synthesized by sensory neurons, the spinal cord, keratinocytes, and endothelial cells, and it exerts its biological effects via ET-A and ET-B receptors [218]. Enhanced ET-1 expression has been documented in the skin of individuals with AD, particularly in association with interleukin-25 (IL-25) activity [105]. In keratinocytes, IL-25 upregulates ET-1, which in turn further elevates IL-25 levels, establishing a positive feedback loop [219]. Elevated epidermal ET-1 levels have also been found in psoriasis, and similar trends are reported in prurigo nodularis, pointing to involvement of the IL-17A/ET-1 pathway in its pathogenesis [107]. Collectively, these findings support a pruritogenic function for ET-1 in itchy skin disorders and highlight its potential as a therapeutic target, particularly in the context of psoriasis [106].

#### Glutamate

Glutamate is a fundamental excitatory neurotransmitter in itch pathways. Although early work suggested that GRP itself might act as the primary transmitter, subsequent studies showed that GRP-responsive neurons in the superficial dorsal horn primarily receive C-fiber inputs mediated by glutamate through AMPA/kainate receptors [220]. In line with this, optogenetic activation of MRGPRA_3_^+^ afferents, which express the vesicular glutamate transporter VGLUT2, induces scratching behavior in a glutamate-dependent manner [221]. These findings establish glutamate as indispensable for transmitting pruriceptive input from primary afferents to spinal circuits, contrary to earlier reports that considered it dispensable [222].

Beyond classical synaptic transmission, glutamate also modulates itch via neuroimmune interactions. Recent work identified a subset of MrgprD^+^ sensory neurons that release glutamate to directly suppress mast cell hyperreactivity through KA2/GluR2 receptors. This tonic inhibition maintains mast cells in a quiescent state and prevents excessive skin inflammation in models of irritant dermatitis, allergic hypersensitivity, and infection [223]. Together, these findings position glutamate as both a primary excitatory transmitter and an immunomodulatory regulator in itch.

## Glial cell regulation of itch circuits under itch conditions

Recent findings have highlighted the involvement of glial cells in regulating the spinal itch circuit and promoting itch. Specifically, astrocytes and microglia are activated in the spinal cord and contribute to itch in various skin inflammation models. In addition, peripheral glial cells, including SGCs in sensory ganglia and Schwann cells along peripheral nerves, have emerged as important modulators of itch processing. Understanding itch-related glial–neuronal communication is essential for effective clinical management of itch symptoms.

### Inflammatory mediators

**Fig. 3:**
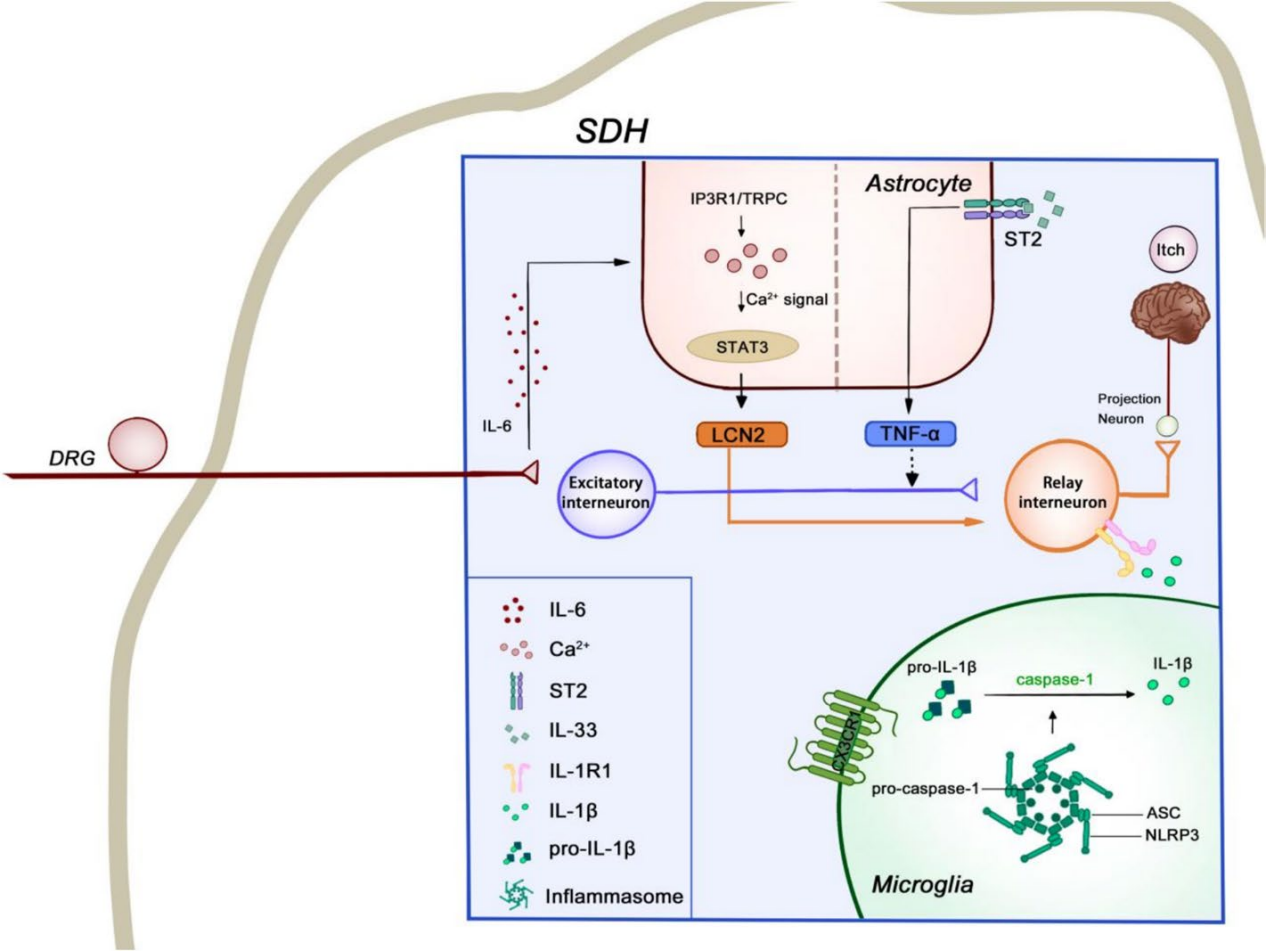
Glial cell modulation of spinal itch circuits under pruritic conditions. Within the superficial dorsal horn (SDH), astrocytes and microglia modulate the excitatory relay from primary afferents to spinal interneurons and projection neurons. Astrocytes are activated by IL-6 arriving from DRG neurons, engaging STAT3 signaling and releasing LCN2, thereby facilitating the interneuron relay. In parallel, IL-33 acting at astrocytic ST2 (IL-33R) increases TNF-α; the dashed arrow indicates a facilitatory effect on the relay with undefined cellular targets in this schematic. Microglia, activated, e.g., via CX3CL1–CX3CR1 signaling, assemble the NLRP3–ASC–pro-caspase-1 inflammasome, generating active caspase-1 and mature IL-1β. Released IL-1β acts on IL-1R1 expressed by relay interneurons, enhancing transmission toward projection neurons and the ascending pathway. The diagram is illustrative rather than exhaustive. This figure was created with Adobe Photoshop

Beyond sensing inflammatory cues through specialized receptors, sensory neurons also directly express and secrete pruritogenic mediators that actively promote itch. Key inflammatory mediators produced by sensory neurons include interleukin-6 (IL-6), CCL2, and C–X–C motif chemokine ligand 12 (CXCL12), with IL-6 exerting central effects in the spinal cord, while CCL2 and CXCL12 primarily act peripherally in the skin. In a mouse model of contact dermatitis-induced itch, IL-6 expression was upregulated in DRG neurons. DRG-derived IL-6 triggered persistent activation of spinal astrocytic STAT3 and subsequent lipocalin-2 (LCN2) production, which enhanced the excitability of GRPR^+^ itch neurons in the SDH [224]. However, in the ACD model, CCL2 and CXCL12 were upregulated in nociceptors and acted through their respective receptors (CCL2 via CCR2 and CXCL12 via CXCR4) in autocrine or paracrine manners to mediate inflammatory skin itch [85, 86] (Fig. 3).

### Spinal astrocyte activation and itch

In multiple itch models—including NC/Nga atopic dermatitis, DNFB/DCP-induced contact dermatitis, and the AEW dry-skin model—spinal astrocytes exhibit reactive changes with increased GFAP expression, and astrocytic STAT3–LCN2 signaling contributes to pruritic sensitization [225–231]. The transcription factor STAT3 plays a pivotal role in the reactive process of astrocytes [232]. STAT3 is transiently activated via phosphorylation by Janus kinases (JAK) following stimulation of cytokine receptors [233]. Multiple ligand–receptor pathways converge on the STAT3 signaling cascade, which functions as a transcriptional hub in astrocytes to drive reactive gliosis and promote pruriceptive transmission [224, 234, 235]. Pharmacological inhibition or genetic disruption of STAT3 effectively suppressed reactive astrogliosis in the SDH of mice, identifying STAT3 signaling as a key mechanism underlying itch [226]. Notably, STAT3-induced astrogliosis was implicated exclusively in pathological chronic itch, with no involvement in acute itch induced by pruritogens [226, 236–238]. A study in mice further revealed that astrocytic STAT3 activation in itch models is driven by DRG-derived IL-6 stimulation through IP3 receptor type 1 (IP3R1)-transient receptor potential canonical (TRPC)-mediated Ca^2+^ influx. This Ca^2+^ influx sustains persistent STAT3 activation, which, in turn, induces astrocytic LCN2 production [224]. While LCN2 alone does not evoke itch behavior, it sensitizes GRPR^+^ neuron excitability when coapplied with GRP, thereby exacerbating itch in mouse contact dermatitis models by establishing spinal central sensitization of the itch circuit [224, 227]. The stable specialized pro-resolving mediator analog, mimetic 3-oxa-PD1n-3 docosapentaenoic acid, has been shown to alleviate acute and chronic itch via suppressing LCN2 secretion from astrocytes in mice [239, 240].

In addition to LCN2, astrocytic tumor necrosis factor-α (TNF-α) also enhances GRP/GRPR signaling in itch. Astrocytic production of TNF-α is driven by activation of the ST2 receptor, which responds to IL-33 released by both oligodendrocytes and astrocytes. In the DNFB-induced mouse ACD model, upregulation of IL-33 and astrocytic ST2 expression enhances this proinflammatory signaling, further amplifying TNF-α synthesis [228].

In the mouse AEW-induced dry skin model, TLR4 expression is elevated in spinal astrocytes, contributing to astrocyte activation, chronic itch, and alloknesis, while having no effect on acute itch responses [89, 230]. Its contribution to itch circuit central sensitization could be further investigated in the future. In summary, astrocytes contribute to itch central sensitization by facilitating GRP-GRPR transmission, highlighting their pivotal role in the pathogenesis of chronic itch.

### Spinal microglia activation and itch

Microglia, the innate immune cells of the CNS, share functional similarities with macrophages and have recently been implicated in itch processing. Yang and colleagues showed that selective ablation of SDH microglia markedly attenuated both histamine-dependent itch (elicited by histamine and compound 48/80) and histamine-independent itch (induced by chloroquine) [241]. However, microglia contribute to histamine-dependent and histamine-independent itch signaling through distinct mechanisms. In histamine-dependent itch, the C–X3–C motif chemokine ligand 1 (CX3CL1)/C–X3–C motif chemokine receptor 1 (CX3CR1) signaling pathway appears to be a key mediator of microglial activation, which in turn enhances neuronal activity. In contrast, the downstream mechanisms underlying microglial involvement in histamine-independent itch remain unresolved [241, 242].

Notably, recent studies have also highlighted the role of microglia in pathological itch. Saika et al. revealed that TRPV1^+^ nociceptors are essential for microglial activation in a mouse psoriasis model [243]. Liu et al. demonstrated that upregulation of the microglial marker ionized calcium-binding adapter molecule 1 (Iba1) is observed in the SDH of mouse itch models, including ACD, psoriasis, dry skin, and AD. This microglial activation is accompanied by NLRP3 inflammasome assembly and elevated IL-1β production. GRPR^+^ neurons, essential for itch signaling, express the type 1 IL-1 receptor (IL-1R1) and are spatially adjacent to IL-1β^+^ microglia; mechanistically, microglia potentiate GRPR^+^ neuron excitability via the NLRP3/caspase-1/IL-1β/IL-1R1 axis, highlighting a key neuroimmune pathway in itch [244].

In a mouse psoriasis model, SDH expression of PD-1 is markedly elevated. Intrathecal administration of PD-L1 attenuates SDH microglial activation and relieves itch in both imiquimod-induced psoriasis and DNFB-induced mouse dermatitis models, though the precise expression pattern of PD-1 in microglia and its mechanistic role in regulating their activation during itch remain unresolved [245, 246]. Immune checkpoint blockade targeting programmed cell death protein 1 (PD-1) or its ligand PD-L1, commonly used in cancer immunotherapy, has been linked to the development of itch as an adverse effect [247]. It is also noteworthy that patients with neuropathic pain may also experience concurrent neuropathic itch. In nerve injury models, microglial activation may contribute to neuropathic pain and itch through distinct intracellular signaling pathways and cytokine profiles [248] (Fig. 3). 

### Peripheral glial cells and itch

Sensory neurons mediating itch reside in DRG and TG. Their pseudounipolar axons bifurcate to innervate peripheral dermatomes and project centrally to the spinal cord. DRG and TG contain satellite glial cells (SGCs) and Schwann cells as the main glial types. Schwann cells are essential for neuronal survival during development and promote regeneration/functional recovery in damaged nerves [249]. SGCs, the predominant glial cells in sensory ganglia, play key roles in sensory function and potentially in pruritus pathogenesis [250]. Studies reveal that SGCs can be directly activated by pruritogenic mediators such as lysophosphatidic acid (LPA), suggesting a link between peripheral neurons and glial cells that might serve as an important mechanism for conditions such as cholestatic pruritus [251]. Beyond the DRG, the role of SGCs has also been investigated in the TG, where the activation marker GFAP was significantly increased in a model of atopic dermatitis, and blocking SGC gap junctions reduced scratching behavior [252]. Additionally, the complement system within SGCs has been identified as potentially playing an essential role in the formation of pruritic sensations, indicating an important link between itch and SGCs in the trigeminal perception system [253]. When activated, SGCs release proinflammatory cytokines and adenosine triphosphate (ATP), which may sensitize primary sensory neurons and enhance their excitability, thereby facilitating itch transmission [252].

Schwann cell dysfunction associates with persistent pruritus: hydroxyethyl starch injection induces severe itch via its accumulation in myelinated/demyelinated Schwann cells, though the underlying molecular mechanisms remain inadequately elucidated in both human patients and mouse models [254, 255]. Recently identified cutaneous Schwann cells sense noxious stimuli and mediate mechanical pain; their potential involvement in itch transmission warrants investigation, offering new perspectives [256].

Lysophosphatidic acid (LPA), a bioactive lipid elevated in cholestatic/hepatic pruritus correlating with severity and treatment response, activates SGCs in DRG cultures [257]. This SGC activation negatively correlates with neuronal transient receptor potential channel responses [251]. Schwann cells also respond to LPA, suggesting LPA-activated glial–neuronal interactions as a mechanism for cholestatic pruritus [251]. In TG of AD mice, GFAP expression, a marker of SGC activation, is significantly increased; systemic inhibition of gap junctions was associated with reduced scratching, but the contribution of SGCs remains uncertain [252]. Complement system components in satellite cells may also contribute to itch sensation, indicating important SGC–itch links in trigeminal perception [253]. Collectively, SGCs and Schwann cells represent promising targets for itch therapies, but further research is needed to elucidate the peripheral glial regulation of itch.

## Future perspectives

Itch results from complex interactions between sensory neurons, immune mediators, and glial cells, involving both peripheral and central neuroinflammatory processes. Peripheral mechanisms depend on pruriceptors and associated glial cells that detect and release inflammatory mediators, while central mechanisms involve the activation of spinal astrocytes and microglia, which amplify itch signaling via cytokine release and activation of signaling pathways such as STAT3 and TLR4. Together, these processes collectively drive the transition from acute to chronic itch, as observed in conditions such as atopic dermatitis, psoriasis, and allergic contact dermatitis. Despite significant advances, significant gaps remain in our understanding of how specific neuroimmune pathways sustain itch. For instance, the molecular mediators released by glial cells and their downstream effects on sensory neurons require further elucidation. Moreover, how peripheral and central itch mechanisms interact to perpetuate the itch–scratch cycle is not fully understood. Advanced genetic and pharmacological tools targeting neuroimmune signaling will be instrumental in addressing these gaps.

Looking ahead, future research should focus on:

1. Identifying novel therapeutic targets: Molecules and pathways, such as STAT3, TLR4, and TRPV1, represent promising candidates for intervention. Exploring their roles in specific itch subtypes could enable personalized treatments.
2. Mechanistic studies on neuroimmune crosstalk: Understanding the bidirectional communication between neurons and immune cells, including mast cells, T-cells, and glial cells, is essential for unraveling the complexity of itch.
3. Exploring glial-specific interventions: Targeting astrocytes and microglia to disrupt central sensitization mechanisms holds potential for managing itch without systemic immunosuppression.
4. Translational approaches: Bridging preclinical findings with clinical applications, including biologics and small-molecule inhibitors, will be critical for developing effective, safe therapies.

In conclusion, a comprehensive understanding of the neuroinflammatory mechanisms underlying itch offers a pathway to innovative and effective treatments. By addressing the interplay between immune and neuronal systems, future research holds the promise of improving quality of life for patients suffering from debilitating itch.

## Data Availability

No datasets were generated or analyzed during the current study.

## Abbreviations

ACD: Allergic contact dermatitis
ACJ: Allergic conjunctivitis
AD: Atopic dermatitis
AEW: Acetone–ether–water
AIA: Antigen-induced arthritis
ATP: Adenosine triphosphate
BAM8-22: Bovine adrenal medulla 8–22
B5-I: Bhlhb5-dependent inhibitory interneurons
Bhlhb5: Basic helix–loop–helix family member B5
C48/80: Compound 48/80
CCL2: C–C motif chemokine ligand 2
CCR2: C–C motif chemokine receptor 2
CGRP: Calcitonin gene-related peptide
CNS: Central nervous system
CQ: Chloroquine
CXCR3: C–X–C motif chemokine receptor 3
CXCR4: C–X–C motif chemokine receptor 4
CX3CL1: C–X3–C motif chemokine ligand 1
CX3CR1: C–X3–C motif chemokine receptor 1
CXCL10: C–X–C motif chemokine ligand 10
CXCL12: C–X–C motif chemokine ligand 12
DCP: Diphenylcyclopropenone
DRG: Dorsal root ganglia
DNFB: Dinitrofluorobenzene
ERK1/2: Extracellular regulated protein kinases ½
ET-1: Endothelin-1
FcεR: Fc epsilon receptor
FcγR: Fc gamma receptor
GABAergic: Gamma-aminobutyric acidergic
GFAP: Glial fibrillary acidic protein
GPCRs: G protein-coupled receptors
GRP: Gastrin-releasing peptide
GRPR: Gastrin-releasing peptide receptor
HR: Histamine receptor
Iba1: Ionized calcium-binding adapter molecule 1
IgE: Immunoglobulin E
IgE-IC: IgE-immune complexes
IgG: Immunoglobulin G
IL-1β: Interleukin-1β
IL-3: Interleukin-3
IL-4: Interleukin-4
IL-6: Interleukin-6
IL-13: Interleukin-13
IL-25: Interleukin-25
IL-31: Interleukin-31
IL-33: Interleukin-33
IL-1R1: Type 1 IL-1 receptor
IL-4Rα: Interleukin-4 receptor α
IL-13Rα1: Interleukin-13 receptor α1
IL-31Rα: Interleukin-31 receptor α
IL-1RAcP: IL-1 receptor accessory protein
IP3R1: IP3 receptor type 1
JAK: Janus kinase
JNK: C-Jun N-terminal kinase
LCN2: Lipocalin-2
LPA: Lysophosphatidic acid
MrgprA3: Mas-related G protein-coupled receptor member A3
MrgprD: Mas-related G protein-coupled receptor member D
Nav1.8: Nav1.8 sodium channels
NK1R: Neurokinin 1 receptor
NLRP3: Nucleotide-binding domain (NBD), leucine-rich repeat (LRR), and pyrin domain (PYD)-containing protein 3
NMB: Neuromedin B
NP: Nonpeptidergic
OSM: Oncostatin M
OSMRβ: Oncostatin M-specific receptor β
PAMPs: Pathogen-associated molecular patterns
PAR-2: Protease-activated receptor 2
PD-1: Programmed death protein1
PD-L1: Programmed death ligand 1
PEP: Peptidergic
PIC: Poly I:C
PLA2: Phospholipase A2
RNA: Ribonucleic acid
SDH: Spinal dorsal horn
SGCs: Satellite glial cells
SP: Substance P
SST: Somatostatin
ST2: Growth stimulation expressed gene 2
STAT: Signal transducer and activator of transcription
SUMOylation: Small ubiquitin-like modifier modification
TG: Trigeminal ganglia
TLR: Toll-like receptors
TLR4: Toll-like receptor 4
TLR5: Toll-like receptor 5
TNF-α: Tumor necrosis factor-α
TRPA1: Transient receptor potential ankyrin 1
TRPC: Transient receptor potential canonical
TRPC3: Transient receptor potential canonical 3
TRPV1: Transient receptor potential vanilloid 1
TRPV4: Transient receptor potential vanilloid 4
TSLP: Thymic stromal lymphopoietin
TSLPR: Thymic stromal lymphopoietin receptor
Ucn3: Urocortin 3
VGLUT2: Vesicular glutamate transporter 2
